# A serum and cream moisturization regimen outperforms separate use in sustaining hydration, reducing TEWL, and balancing surface lipids over an 8‐h study

**DOI:** 10.1111/ics.70100

**Published:** 2026-03-30

**Authors:** Alexandra M. Maldonado López, Ivan Domicio da Silva Souza

**Affiliations:** ^1^ Good Molecules, LLC Philadelphia Pennsylvania USA

## Abstract

Skin hydration can be achieved through complementary mechanisms in skincare formulations. Humectants retain water, while emollients and occlusives reduce transepidermal water loss (TEWL) by reinforcing the epidermal barrier. Although consumers follow skincare routines, biophysical comparisons of single‐product use versus regimen use across parameters such as hydration, TEWL, and sebum content are limited. Our study measured these parameters in 123 volunteers randomized into four treatment groups: Facial Cleanser (FC) control, Hyaluronic Acid Serum (HAS) group, Moisturizing Cream (MC) group, and Routine comprising HAS followed by MC. Corneometry, TEWL, and sebumetry were measured at baseline, 30 min, and 8 h after treatment application. Corneometry results at 30 min showed that HAS, MC, and Routine increased hydration when compared with FC. At 8 h, only the Routine remained higher than FC. Additionally, TEWL decreased at 30 min for HAS, MC, and the Routine versus FC, whereas at 8 h, only HAS remained lower than FC. We measured sebumetry because skin surface lipids support the epidermal barrier and thus maintain water homeostasis. FC reduced sebum at 30 min due to sebum stripping. However, at 8 h, the Routine's sebum measurements stayed closest to baseline and were lower than HAS and MC individually. Apart from objective measurements, perceived hydration was assessed by a subjective questionnaire. Volunteers who applied the routine reported greater immediate and long‐lasting hydration than those who applied HAS or MC individually. These results suggest that layering the Routine improved hydration durability with barrier‐relevant water‐retention benefits over an 8‐h wear period.

## Graphical abstract

Over 8 h, using a Hyaluronic Acid Serum in combination with a Moisturizing Cream sustained skin hydration more than either product alone, while lowering TEWL and normalizing surface lipids. Volunteers also reported a more immediate and long‐lasting moisturization with the combined regimen. Created in BioRender. Maldonado Lopez, A. (2026) https://BioRender.com/va5iqxl.

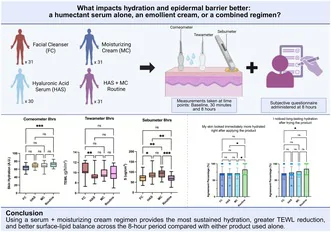

## Résumé

Une hydratation de la peau peut être obtenue grâce à des mécanismes complémentaires dans les formulations pour soins de la peau. Les agents humectants retiennent l'eau, tandis que les agents émollients et occlusifs réduisent la perte d'eau transépidermique (PETE) en renforçant la barrière épidermique. Bien que les consommateurs suivent des routines de soins de la peau, les comparaisons biophysiques entre utilization d'un seul produit et utilization d'une routine à l'égard de paramètres tels que l'hydratation, la PETE ou la teneur en sébum sont limitées. Notre étude a mesuré ces paramètres chez 123 volontaires randomisés dans quatre groupes de traitement: le groupe témoin du nettoyant pour le visage (NV), le groupe du sérum d'acide hyaluronique (SAH), le groupe de la crème hydratante (CH) et une routine composée de SAH puis de CH. L'hydratation par cornéométrie, la PETE et la teneur en sébum par sébumétrie ont été mesurées au début de l'étude, 30 min et 8 heures après l'application du traitement. Les résultats de la cornéométrie après 30 min ont montré que le SAH, la CH et la routine augmentaient l'hydratation par rapport au NV. Après 8 heures, seule la routine restait supérieure au NV. En outre, la PETE avait diminué après 30 min pour le SAH, la CH et la routine par rapport au NV, tandis qu'après 8 heures, seul le SAH restait inférieur au NV. Nous avons mesuré la sébumétrie car les lipides à la surface de la peau favorisent la barrière épidermique et maintiennent ainsi l'homéostasie de l'eau. Le NV a réduit le sébum après 30 min en raison d'un décapage de sébum. Cependant, après 8 heures, les mesures de sébum de la routine restaient plus proches de la valeur de référence et étaient respectivement inférieures à celles du SAH et de la CH. Outre les mesures objectives, l'hydratation perçue a été évaluée par un questionnaire subjectif. Les volontaires ayant appliqué la routine ont rapporté une hydratation immédiate et durable plus importante que ceux ayant respectivement appliqué le SAH ou la CH. Ces résultats suggèrent que l'application de la routine a amélioré la durabilité de l'hydratation avec des avantages en termes de rétention d'eau liés à la barrière sur une période de 8 heures.

## INTRODUCTION

The epidermis' primary function is to act as a barrier between the organism's internal environment and external stressors. The stratum corneum (SC) of the epidermis constitutes the principal physical barrier, protecting against chemical, microbial, and mechanical insults while simultaneously regulating transepidermal water loss (TEWL) [1]. Structurally, the SC is described as the ‘bricks and mortar’ model, in which terminally differentiated corneocytes are embedded within a lipid‐rich intercellular matrix [2, 3, 4] that maintains the skin's water homeostasis. Disruption of the SC impairs skin barrier function and compromises skin hydration, which in part drives facial ageing [5, 6].

Epidermal barrier function is commonly evaluated using biophysical parameters that reflect both structural and functional aspects of the SC, most notably hydration and TEWL [7, 8]. Hydration measurements, in the form of corneometry, reflect the water content of the outermost epidermal layers and indirectly inform on barrier integrity [8, 9]. In contrast, TEWL measures the passive diffusion of water through the SC with elevated values associated with impaired skin barrier and dryness [7, 8]. Consequently, TEWL has become a standard parameter in dermatological and cosmetic research for assessing barrier performance [7]. Skin hydration is typically achieved by two complementary mechanisms: (1) humectants that attract water to increase SC water content, and (2) emollients and/or occlusives that form a hydrophobic film to reduce evaporation and reinforce the skin barrier [5, 10]. A well‐characterized humectant, hyaluronic acid or its salt form, promotes a rapid increase of hydration through its water‐binding properties [5, 10]. On the other hand, emollients such as moisturizing creams (MCs) enhance hydration by supporting SC lipids, thereby reducing TEWL over time [10].

Skin surface lipids also play a crucial role in supporting the epidermal barrier and maintaining water homeostasis [11, 12]. Therefore, it has been proposed that the balance between superficial skin lipids and the SC's water content, often referred to as the water–sebum system, serves as an indicator of overall skin health [11, 13] and alterations to this balance can disrupt barrier integrity and thus compromise skin hydration. Consequently, sebum content should be frequently included in clinical evaluations alongside skin hydration and TEWL [11, 12].

Together, these non‐invasive methods enable a comprehensive assessment of skin barrier integrity and hydration. Hence, the following clinical study evaluates the effects of three skincare routines on corneometry, TEWL, sebum content, and perceived hydration. The three routines comprise a Face Cleanser used in tandem with either a hyaluronic acid serum (HAS), a MC, or in a regimen with both the HAS and MC.

## METHODS

### Study design

This trial was an open‐label, brand‐blinded, randomized, regimen‐controlled study evaluating the efficacy of cosmetic products in improving skin hydration as determined by instrumental and subjective analysis. The protocol complied with the guidelines of the Declaration of Helsinki. It was approved by the Research Ethics Committee (REC) at the Universidade São Francisco, SP, Brazil. The research protocol, CAAE 89234625.5.0000.5514, was approved by the REC under review 7.639.604, issued on June 13th, 2025. All volunteers signed a written informed consent before initiating the study.

Healthy volunteers of legal age with a Fitzpatrick skin type (FST) between I and VI, and of dry or normal facial skin type, who were willing to comply with the procedures, methods, evaluations, and test product usage described in the written informed consent, were included in the study. Volunteers were excluded if they (1) were pregnant, had risk of pregnancy or were lactating during the study; (2) had skin marks in the region of the study that interfered in the evaluation (vascular malformations, scars, large quantities of ephelides or nevi, sunburn); (3) presented history of atopy or of allergic reactions, irritation or intense sensations of discomfort to products of topical use: cosmetics or drugs; (4) presented dermatoses or active skin lesions (local and/or disseminated) in the study area; and (5) used non‐steroidal anti‐inflammatory drugs, corticosteroids, antihistamines, photosensitizers, or immunosuppressants 2 weeks before the start of the study; (6) were treated with vitamin A acid and/or its derivatives up to 1 month before the study; (7) applied any topical/dermatological products on the study area 3–5 days before the start of the study; (8) received aesthetic or dermatological treatment up to 4 weeks before the start of the study; (9) if there had been changes in hormonal treatment (medication and/or dosage) within the last 3 months; (10) decompensated endocrinopathies; (11) had intense sun exposure or tanning session within 15 days before the initial evaluation; or (12) any condition that in the opinion of the clinical staff, could compromise the study.

A 124 healthy volunteers, aged 18–60 years, male and female, who signed informed consent, participated in the study. All volunteers were instructed to discontinue use of any cosmetic products on their faces (the application site), including serums, lotions, and moisturizers, for 3–5 days prior to the first day of the study. The volunteers were randomly assigned to four study groups. All groups initially used a Facial Cleanser (FC), with one group designated as the control group, which used only the FC. After using the FC, one group applied only the HAS, while another group applied only the MC. The fourth group followed the complete skincare Routine, applying FC, then HAS, and lastly MC, with about 1 min between products. Products were purchased from authorized online retailers, and their ingredient lists are provided in Table 1. Of the 124 research volunteers selected for the study, 123 completed it (Table 2). One volunteer from the HAS group withdrew from the study for personal reasons unrelated to the study. During the clinical visit, the volunteers remained in a climate‐controlled room for at least 30 min before the start of the evaluations and throughout data collection (22°C ± 2°C and 55% ± 5% relative humidity).

**TABLE 1:** Test products.

**Face Cleanser**
Ingredients: Water, Glycerin, Cetearyl Alcohol, Panthenol, Niacinamide, Pantolactone, Xanthan Gum, Sodium Cocoyl Isethionate, Sodium Benzoate, Citric Acid
**Hyaluronic Acid Serum**
Ingredients: Water, Propanediol, Glycerin, Sodium Hyaluronate (1.0%, per label), Hydroxyacetophenone, Sodium Citrate, Hydrolysed Hyaluronic Acid, Citric Acid, Ethylhexylglycerin, Tocopherol
**Moisturizing Cream**
Ingredients: Water, Glycerin, Cetearyl Alcohol, Caprylic/Capric Triglyceride, Cetyl Alcohol, Ceteareth‐20, Petrolatum, Potassium Phosphate, Ceramide NP, Ceramide AP, Ceramide EOP, Carbomer, Dimethicone, Behentrimonium Methosulfate, Sodium Lauroyl Lactylate, Sodium Hyaluronate, Cholesterol, Phenoxyethanol, Disodium EDTA, Dipotassium Phosphate, Tocopherol, Phytosphingosine, Xanthan Gum, Ethylhexyglycerin

**TABLE 2:** Clinical study demographics.

Group	Age (mean ± SD)	Gender	FST	Facial skin type
FC	44 ± 13 yo	87% female	0% II	35% dry
			71% III	
		13% male	13% IV	65% normal
			6% V	
			10% VI	
HAS	44 ± 11 yo	70% female	10% II	20% dry
			57% III	
		30% male	16% IV	80% normal
			10% V	
			7% VI	
MC	42 ± 12 yo	77% female	0% II	32% dry
			58% III	
		23% male	23% IV	68% normal
			13% V	
			6% VI	
Routine	43 ± 11 yo	87% female	0% II	29% dry
			65% III	
		13% male	13% IV	71% normal
			19% V	
			3% VI	

Abbreviations: FC, facial cleanser control; FST, fitzpatrick skin type; HAS, hyaluronic acid serum; MC, moisturizing cream; Routine, hyaluronic acid serum and moisturizing cream combination; yo, years old.

Ingredients are listed by their INCI (International Nomenclature of Cosmetic Ingredients) names.

Randomized corneometry, TEWL, and sebum measurements were taken on either the left or right malar area of the research volunteers. The assessments were performed at the beginning of the study (baseline), after 30 min, and after 8 h of the treatment application (Figure 1). At the end of the study, the volunteers completed a subjective questionnaire to express their opinions on the test samples.

**FIGURE 1:**
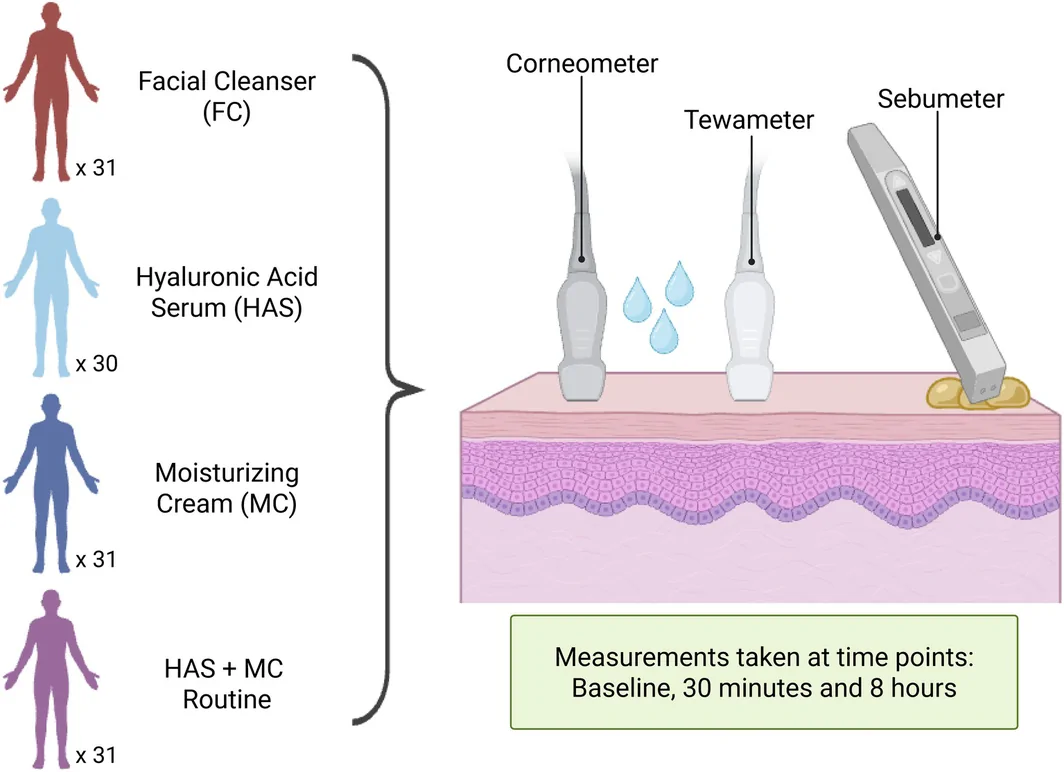
Clinical study groups and assessment schedule. Volunteers were randomized into one of four study groups: Facial Cleanser (FC; *n* = 31), Hyaluronic Acid Serum (HAS; *n* = 30), Moisturizing Cream (MC; *n* = 31), or a combined HAS + MC Routine (*n* = 31). Skin hydration, TEWL, and sebum content levels were measured using a Corneometer®, Tewameter, and Sebumeter, respectively, at baseline, 30 min, and 8 h.

### Skin hydration measurements

Skin hydration was measured with a Corneometer® CM 825 (Courage + Khazaka Electronic, Germany) coupled to the Multi Probe Adapter dual MPA 6 (Courage + Khazaka Electronic, Germany) by determining the mean values of capacitance (h), the variation in capacitance measurements (Δh), and the percentage of increase in skin hydration (%H) using Equations (1 and 2).

(1) Δh=hti–ht0

Variation in capacitance measurements (Δh), where hti = mean values of capacitance obtained after 30 min and 8 h of the treatment application; ht0 = mean values of capacitance obtained at the beginning of the study.

(2) %H=100×Δh/ht0

Percentage of increase in skin hydration provided by the treatment (%H), where Δh = variation in capacitance measurements for the treatment after 30 min and 8 h of the treatment application; ht0 = mean values of capacitance obtained at the beginning of the study. As skin capacitance increases, %H increases, which is a positive outcome.

### Transepidermal water loss measurements (TEWL)

TEWL was measured with a Tewameter® TM HEX probe (Courage + Khazaka Electronic, Germany) coupled to the Base Multi Probe Adapter MPA 6 (Courage + Khazaka Electronic, Germany) by determining the mean values of TEWL (e), the variation in TEWL measurements (Δe), and the percentage of water loss (%E) using Equations (3 and 4).

(3) Δe=et0–eti

Variation in transepidermal water loss measurements (Δe), where eti = mean values of TEWL obtained after 30 min and 8 h of the treatment application; et0 = mean values of TEWL obtained at the beginning of the study.

(4) %E=−100×Δe/et0

Percentage of water loss (%E), where Δe = variation in TEWL measurements calculated after 30 min and 8 h of the treatment application; et0 = mean values of TEWL obtained at the beginning of the study. When greater evaporation or water loss is observed, %E increases, which is a negative outcome.

### Sebum measurements

Sebum content was measured with a Sebumeter® SM 815 equipment (Courage + Khazaka Electronic, Germany) coupled to the Base Multi Probe Adapter MPA 580 (Courage + Khazaka Electronic, Germany) by determining the mean values of sebaceous secretion (s) and the percent decrease in skin oiliness (%DO) using Equation (5).

(5) $$\%\mathrm{DO}=100\times \left(\mathrm{st}0-\mathrm{sti}\right)/\mathrm{st}0$$

Percentage of decrease in skin oiliness (%DO), where s = mean values of sebaceous secretion; ti = values obtained after 30 min and 8 h; t0 = values obtained at the beginning of the study. When higher levels of skin oiliness are observed, %DO decreases, which is a negative outcome.

### Subjective questionnaire

The volunteers completed a subjective questionnaire to express their opinions on the test products. The perceived efficacy was evaluated using a 4‐point Likert scale. Scores 1 and 2 (rejection: strongly disagree and disagree, respectively) indicated that the volunteer viewed the attribute negatively. In contrast, scores 3 and 4 (acceptance: agree and strongly agree, respectively) showed a positive perception. To better visualize the mean results, these scores were converted to percentages of agreement. Score 1 represents 0% agreement, score 2 represents 33.3%, score 3 represents 66.67%, and score 4 represents 100% agreement.

### Statistical analysis

Statistical analyses and graphs were produced using GraphPad Prism 10 (GraphPad, Boston, Massachusetts, USA), unless otherwise noted. A one‐factor analysis of variance (ANOVA) was conducted, followed by post hoc Tukey tests for within‐ and between‐group comparisons at baseline and subsequent time points. Analyses considered a 95% confidence interval for all values obtained for the treatments. A *p*‐adjusted value of <0.05 was considered statistically significant for comparisons to the baseline. The questionnaire responses were analysed using the Kruskal–Wallis test with Dunn's multiple‐comparison test; *p*‐adjusted values <0.05 were considered statistically significant. Baseline demographic characteristics (sex, age, FST, facial skin type) were compared between groups using Pearson's Chi‐square test (*χ* ^2^) with Bonferroni corrections performed in IBM SPSS Statistics, version 30.0.0.0 (IBM Corp., Armonk, New York, USA). When more than 20% of tabulated cells had an expected count less than 5, we reported the *χ* ^2^ Likelihood ratio's *p*‐value, which is less affected by sparse cells.

## RESULTS AND DISCUSSION

A 123 volunteers were randomly assigned to four treatment groups (Figure 1): a control Facial Cleanser (FC), Hyaluronic Acid Serum (HAS), Moisturizing Cream (MC), and HAS + MC combination routine (Routine). The study volunteers reported no adverse events and no significant differences were observed for age (*χ* ^2^ = 14.906, df = 15, *p* = 0.267), gender (*χ* ^2^ = 4.011, df = 3, *p* = 0.260), Fitzpatrick Skin Type (*χ* ^2^ = 14.396, df = 12, *p* = 0.331), or Facial Skin Type (*χ* ^2^ = 1.958, df = 3, *p* = 0.581) amongst the groups at baseline (Table 1). The treatments were evaluated for their effects on physiological skin parameters, such as skin hydration, TEWL, and sebum production.

Corneometer measurements demonstrate changes in capacitance that correspond to the amount of water in the skin. These measurements are converted into arbitrary hydration units, ranging from 0 to 120 A.U., with 0 units indicating very dry skin and 120 units indicating very hydrated skin. We first conducted within‐group analyses to examine the hydration effects of each treatment over 8 h (Figure 2a–d). After 30 min of treatment application (HAS, MC, Routine) or control (FC), skin hydration increased significantly (*p* < 0.05) relative to the respective baseline corneometer measurements. However, at the 8‐h mark, only the treatments MC and Routine remained significantly higher than their baseline (Figure 2a–d). Between‐group analyses were also conducted to evaluate the differences between treatments in hydration at each time point (Figure 2e–g). When comparing the treatments against each other and against the control, baseline corneometer measurements were not significantly different (Figure 2e), validating the homogeneity of the groups before intervention. At 30 min, all treatments (HAS, MC, and Routine) showed significantly greater hydration than the FC control (17.8%, 17.7%, and 20.0%, respectively, with an adjusted *p*‐value [*p*‐adj] < 0.0001; Figure 2f). Notably, at 8 h, only the Routine exhibited a significant increase in skin hydration compared with the control (11.4%, *p*‐adj = 0.004; Figure 2g). These corneometer results indicate that although using HAS or MC individually increases skin hydration immediately after application, volunteers using the Routine show significant improvements at 30 min and 8 h post‐application. Additionally, we measured percent hydration (%H) across time points for the different treatment groups (Figure 2h,i). At the 30‐min mark, all treatments showed a significant increase in %H relative to the control (Figure 2h). Interestingly, at the 8‐h mark, HAS did not show a significant increase relative to the control FC, whereas the MC and Routine groups showed an increase relative to FC. Furthermore, MC and Routine treatments showed significantly higher hydration percentage than HAS at 8 h post‐application (Figure 2i). Using the Routine also demonstrated significantly greater hydration than MC alone at the 8‐h mark (Figure 2i), indicating greater sustained skin hydration when using the Routine in comparison with HAS and MC individually.

**FIGURE 2:**
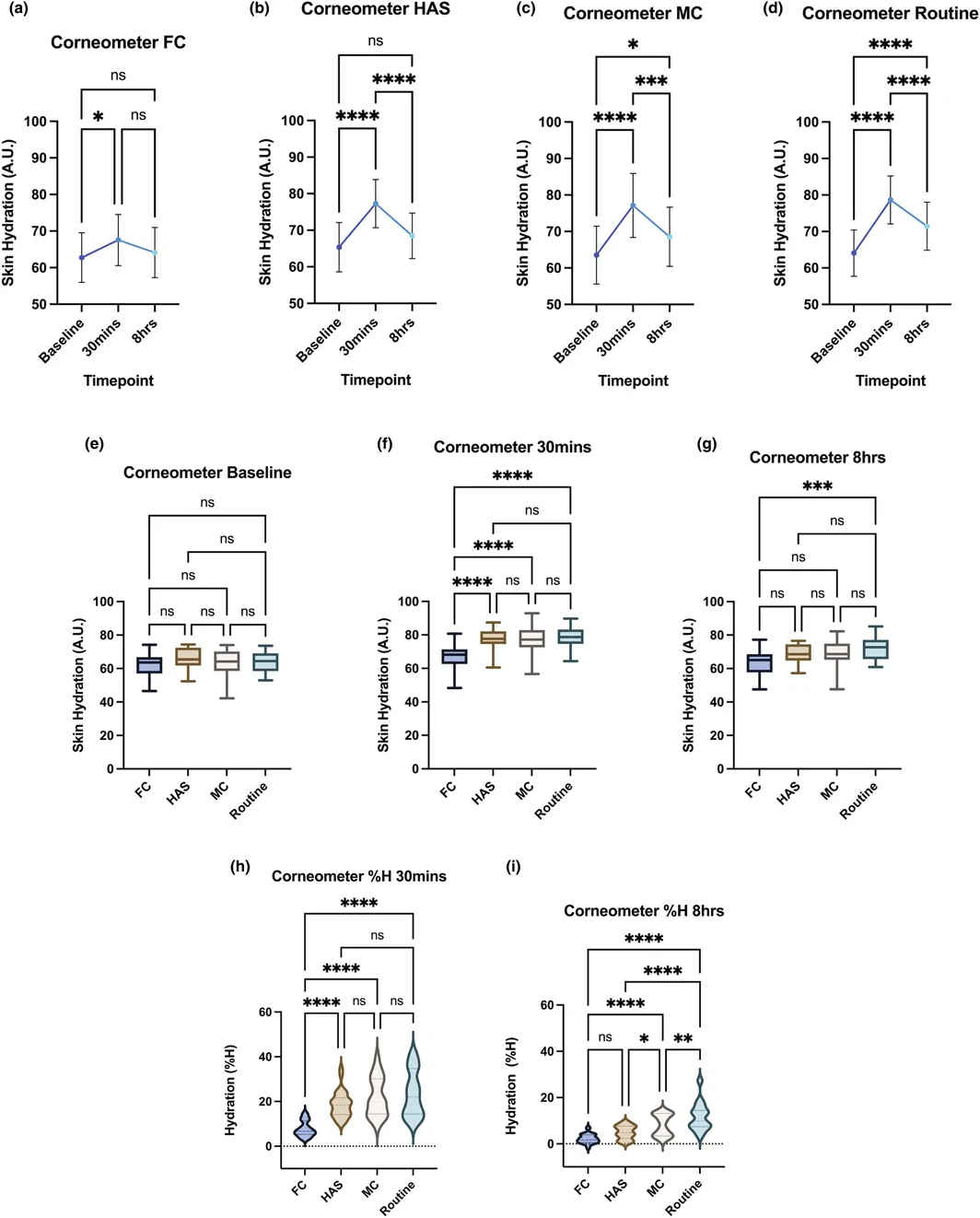
Corneometer‐assessed facial skin hydration across study groups and time points. (a–d) Within‐group skin hydration at baseline, 30 min, and 8 h for the Facial Cleanser (FC) group (a), Hyaluronic Acid Serum (HAS) group (b), Moisturizing Cream (MC) group (c), and Routine group (d). (e–g) Between‐group comparisons of skin hydration at baseline (e), 30 min (f), and 8 h (g). (h, i) Percent change in hydration relative to baseline (%H) at 30 min (h) and 8 h (i). Brackets denote pairwise comparisons; ns indicates not significant; *p < 0.05, **p < 0.01, ***p < 0.001, ****p < 0.0001.

To measure TEWL, a microprocessor analyses the quantity of water lost from inside the body by diffusion across the SC of the epidermis, and expresses the evaporation rate in grams per hour per square metre (g/h/m^2^). The measurements obtained are objective and quantitative, as healthy skin typically exhibits low TEWL values and high levels of aqueous content in the SC. Within‐group Tewameter analyses were conducted to study the amount of water loss after the application of each treatment (Figure 3a–d). After 30 min of treatment application (HAS, MC, and Routine), TEWL decreased significantly from baseline (*p*‐adj < 0.005). This decrease was not observed in the FC control group. However, at the 8‐h mark, only the Routine maintained TEWL levels significantly lower than baseline (Figure 3a–d). When analysing the between‐group TEWL measurements, baseline TEWL did not differ significantly (Figure 3e), validating the homogeneity of the groups before intervention. At the 30‐min time point, all treatments (HAS, MC, and Routine) significantly reduced TEWL compared with the control cleanser, FC (−14.6% with *p*‐adj < 0.001, −10.0% with *p*‐adj = 0.001, and − 14.7% with *p*‐adj < 0.001, respectively; Figure 3f). Remarkably, at 8 h, volunteers who received only HAS had a significant decrease in TEWL compared with the control (−9.6%, *p*‐adj = 0.008; Figure 3g). These Tewameter results suggest that all treatments reduce skin water loss immediately after application, whereas HAS application maintains the high water content at 8 h. Regarding water loss percentage (%E), all treatments showed significant reductions compared with the control group at both the 30‐min and 8‐h time points (Figure 3h,i). Notably, there was no significant difference in %E between the HAS and MC at either time point. However, the Routine showed a significant difference compared with each treatment alone at both time points (Figure 3h,i). These results indicate that all treatments effectively reduce TEWL and help retain water content both immediately after application and after 8 h, with the Routine retaining the most water in the skin.

**FIGURE 3:**
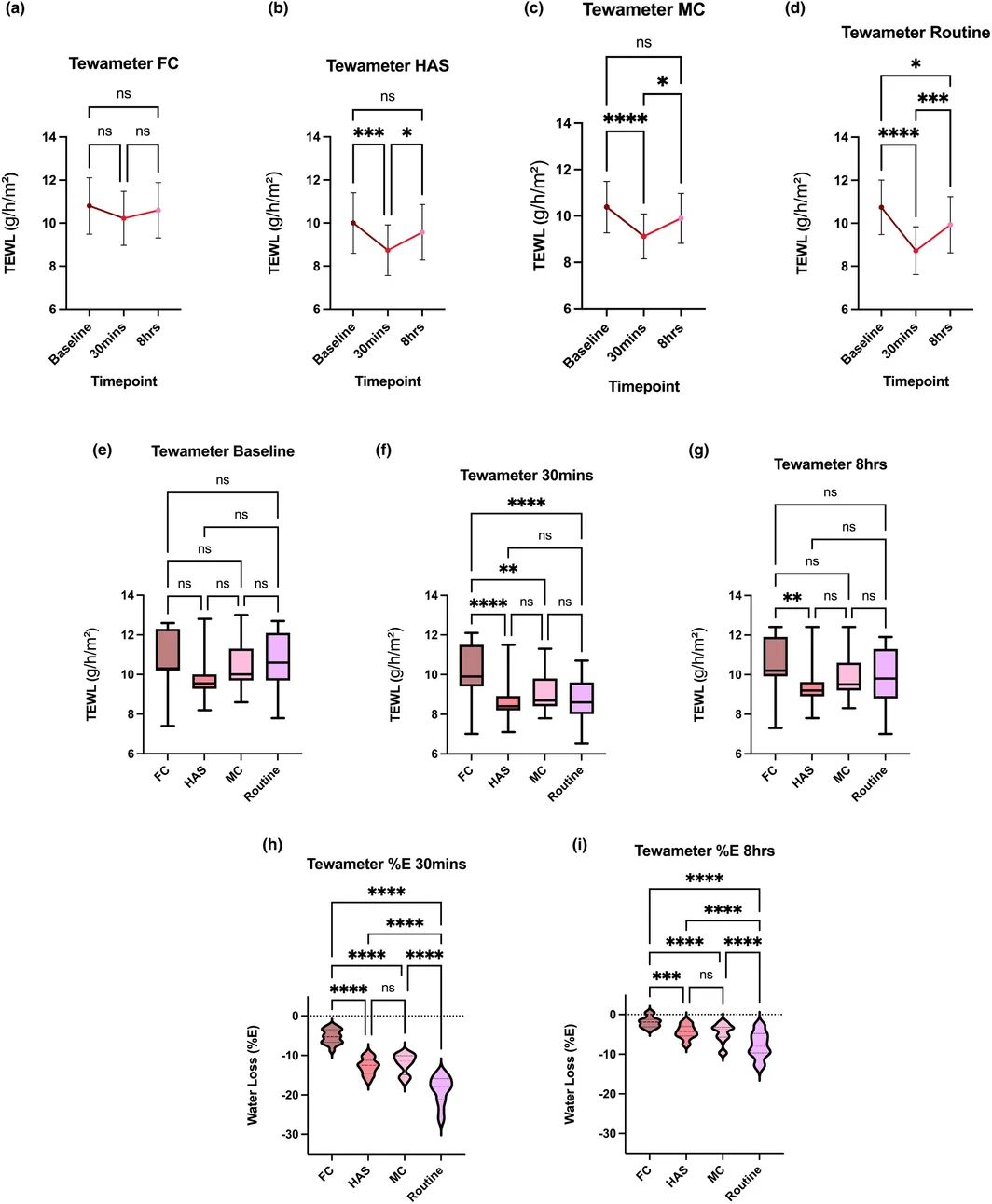
Tewameter‐assessed transepidermal water loss (TEWL) across study groups and time points. (a–d) Within‐group TEWL at baseline, 30 min, and 8 h for the Facial Cleanser (FC) group (a), Hyaluronic Acid Serum (HAS) group (b), Moisturizing Cream (MC) group (c), and Routine group (d). (e–g) Between‐group comparisons of TEWL at baseline (e), 30 min (f), and 8 h (g). (h, i) Percent change in TEWL relative to baseline (%E) at 30 min (h) and 8 h (i). Brackets indicate pairwise comparisons; ns denotes not significant; *p < 0.05, **p < 0.01, ***p < 0.001, ****p < 0.0001.

Sebumeters, unaffected by humidity, measure the amount of sebaceous secretion on a tape by maintaining constant pressure on a single site for 30 s and report results in μg/cm^2^. The amount of sebum produced in the span of 30 min and 8 h after the application of each treatment was analysed within groups (Figure 4a–d). After 30 min of application of the control (FC), we observed a significant decrease in sebum secretion (*p*‐adj < 0.005) compared with baseline, which then normalizes back to baseline levels at 8 h (*p*‐adj > 0.05). This finding is understandable, as cleansers typically remove sebum from the skin [3, 14, 15]. This decrease was not observed with the HAS, MC, and Routine treatments. At the 8‐h mark, the HAS and MC individual treatments showed a significant increase in sebum secretion compared with their respective baselines and post‐30‐min results. Further, the Routine did not show a significant sebum increase at the 30‐min and 8‐h time points in comparison to its baseline, even though it showed a significant increase (*p*‐adj < 0.05) in sebum secretion from the 30‐min to the 8‐h time point (Figure 4a–d). Between‐group analyses revealed no significant difference in baseline Sebumeter measurements (Figure 4e), validating the homogeneity of the groups before intervention. At the 30‐min mark, both HAS and MC treatments alone showed a significant increase in sebum secretion compared with FC (44.2% and 50.6%, respectively, *p*‐adj < 0.0001), whereas the Routine did not (24.5%, *p*‐adj = 0.060). In addition, the Routine group showed significantly less sebum secretion than MC alone at 30 min (−17.3% with a *p*‐adj = 0.039; Figure 4f). At the 8‐h point, Routine was the only treatment that did not exhibit a significant increase in sebum secretion compared with the control. Yet, it showed a significant decrease relative to both HAS and MC treatments (−20.2% with *p*‐adj = 0.006 and − 24.0% with *p*‐adj = 0.0003, respectively; Figure 4g). These results suggest that the combined use of HAS and MC did not increase sebum production on the skin, even after 8 h of use, thus helping maintain baseline sebum levels. In terms of the percentage decrease in skin oiliness (%DO), all treatments (HAS, MC, and Routine) showed significantly lower mean %DO relative to the control FC at the 30‐min mark, suggesting an increase in skin oiliness (Figure 4h). However, at the 8‐h mark, none of the treatments showed a significant decrease in skin oiliness relative to the control or other treatments (Figure 4i). These results indicate that the treatments increase skin oiliness immediately after application, but they do not continue to increase the oiliness throughout the rest of the day. Regardless, limited conclusions can be drawn about sebum production or lipid increases induced by the treatments, as the Sebumeter only measures sebum content on the skin surface.

**FIGURE 4:**
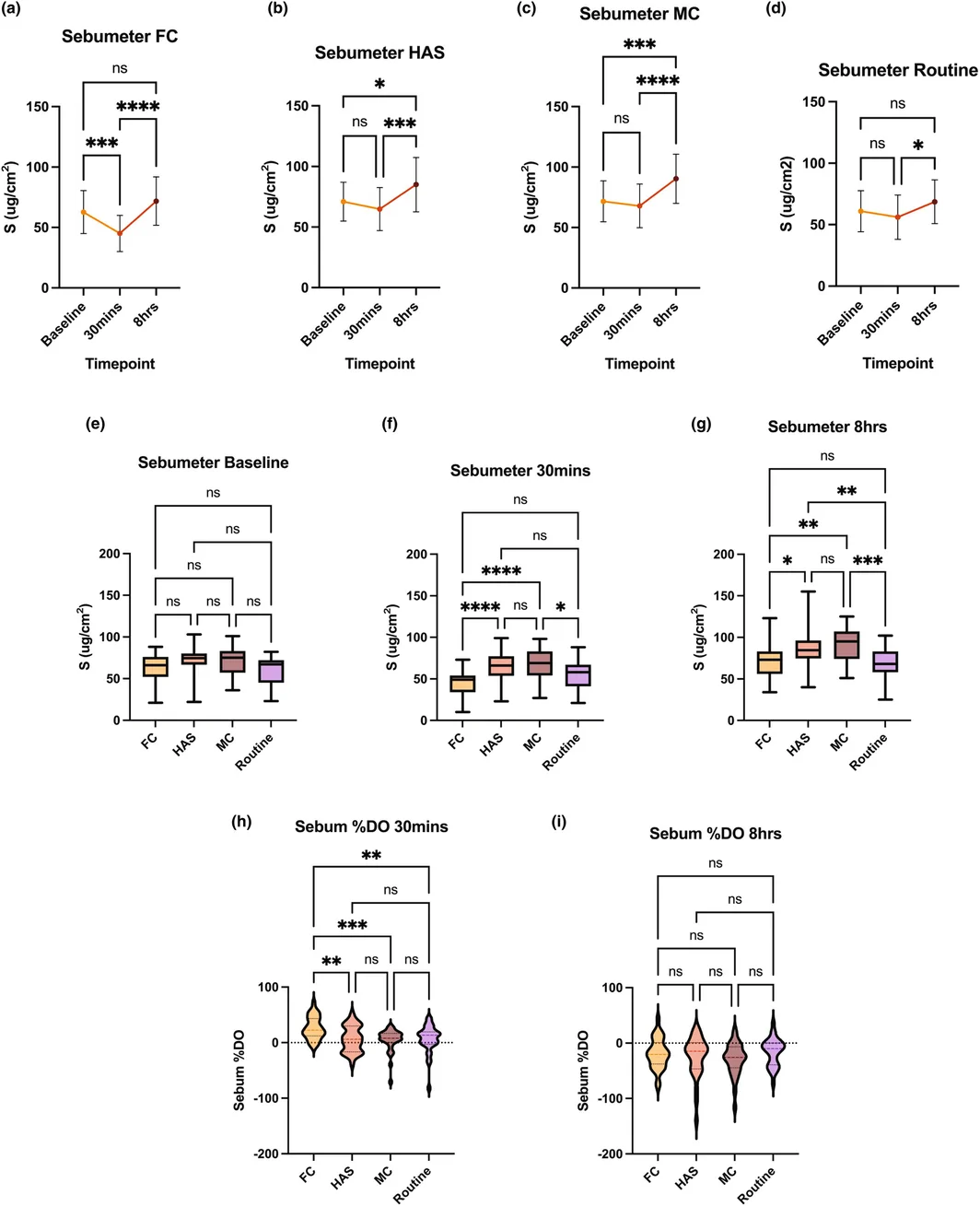
Sebumeter‐assessed facial sebum content levels across study groups and time points. (a–d) Within‐group sebum at baseline, 30 min, and 8 h for the Facial Cleanser (FC) group (a), Hyaluronic Acid Serum (HAS) group (b), Moisturizing Cream (MC) group (c), and Routine group (d). (e–g) Between‐group comparisons of absolute sebum at baseline (e), 30 min (f), and 8 h (g). (h, i) Percent change in sebum relative to baseline (%DO) at 30 min (h) and 8 h (i). Brackets indicate pairwise comparisons; ns denotes not significant; *p < 0.05, **p < 0.01, ***p < 0.001, ****p < 0.0001.

At the end of the study, a subjective questionnaire was applied to assess the volunteers' opinions on the treatments. Volunteers who received the Routine treatment reported a significantly higher percent agreement than those who received HAS or MC treatments alone when asked whether their skin appeared immediately more hydrated after product application (Figure 5a). There was no significant difference between the treatment groups when asked if their skin looked more hydrated at the end of the day (Figure 5b), if their skin felt deeply hydrated at the end of the day (Figure 5c), if their skin looked more plump at the end of the day (Figure 5e), or if their skin felt firmer at the end of the day (Figure 5f). However, the Routine group showed a greater percent agreement compared with the other treatments and the control. Regarding long‐lasting hydration, volunteers using the Routine reported a higher percent agreement than others, but this difference was statistically significant only when compared with the control group (Figure 5d). These subjective insights indicate that although all treatments increased the perception of hydration and skin plumpness and firmness compared with the control, only the Routine provided significantly more hydration immediately after application, a difference that persisted over time.

**FIGURE 5:**
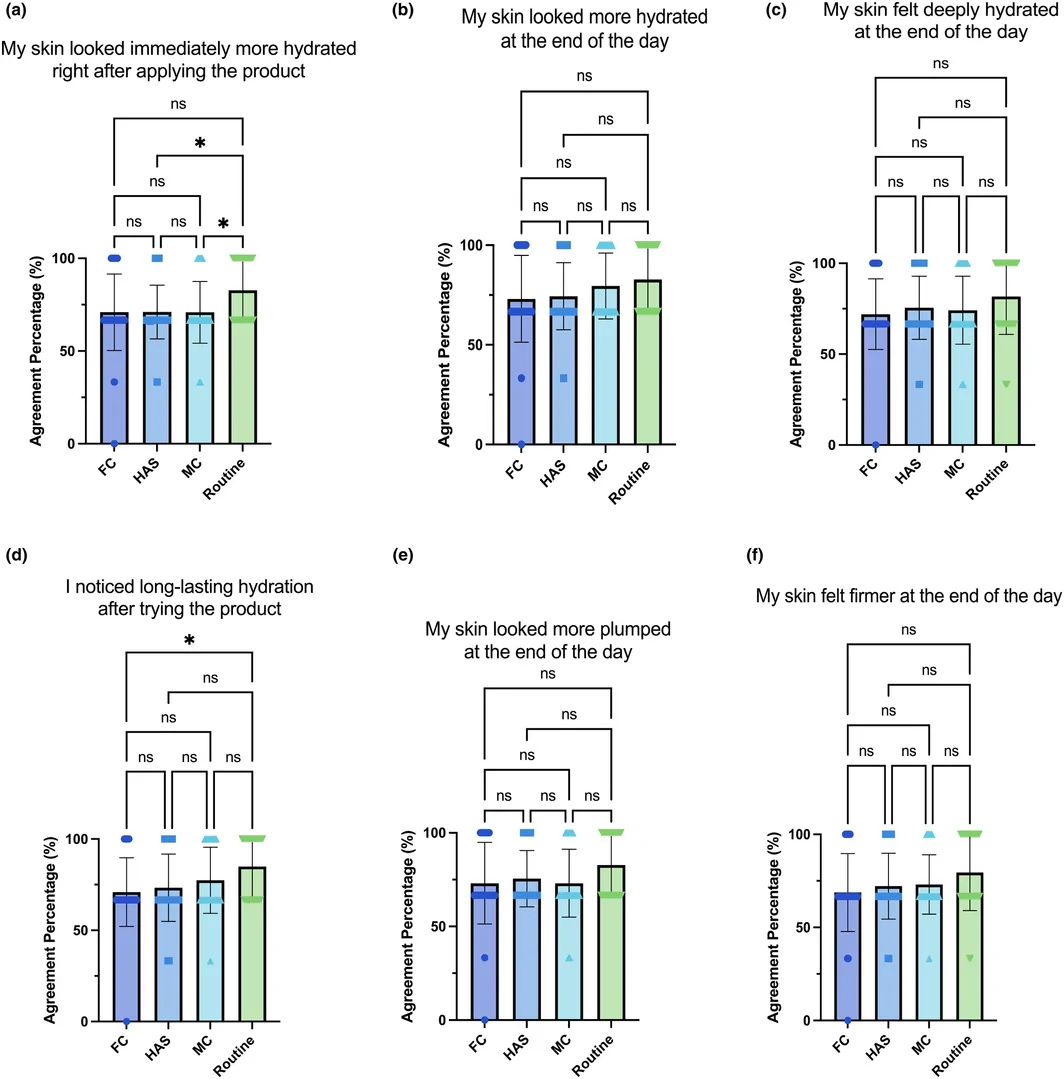
(a–f ) Volunteer‐reported perceptions of skin hydration and feel. Agreement percentages for six subjective questionnaire statements regarding the Facial Cleanser (FC), Hyaluronic Acid Serum (HAS), Moisturizing Cream (MC), and Routine groups were calculated using a 4‐point Likert scale. Scores of 1 and 2 represent ‘strongly disagree’ and ‘disagree’, while scores of 3 and 4 represent ‘agree’ and ‘strongly agree’. These scores were converted to percentages of agreement: 0% for score 1, 33.3% for score 2, 66.67% for score 3, and 100% for score 4. Brackets indicate pairwise comparisons; ‘ns’ means not significant; and statistical significance levels are indicated as *p < 0.05, ns = not significant.

## DISCUSSION

This randomized clinical study assessed the effects of HAS treatment, MC treatment, and their combination (Routine) on three key biophysical parameters associated with skin barrier integrity and hydration. After 30 min of their application, increases in skin hydration and reductions in TEWL were measured across all treatment groups. These findings are consistent with previous clinical studies, which report well‐established correlations between skin hydration and epidermal barrier integrity, and demonstrate that moisturizers rapidly improve skin hydration [16, 17, 18]. However, in our study, differences emerged over time, highlighting formulation‐dependent effects on skin hydration.

The volunteers receiving HAS or MC alone developed a significant increase in short‐term hydration, consistent with the humectant properties of hyaluronic acid or the moisturizing effects of emulsion‐based products [5, 19]. However, HAS alone did not sustain the significant skin hydration after 8 h of its application, supporting prior observations that humectants require complementary occlusion to prolong hydration [10]. When combining HAS and MC into a routine, significantly increased skin hydration was maintained for up to 8 h after their application. TEWL findings further support these corneometer results. While all volunteer groups showed reduced TEWL shortly after treatment, only the volunteers applying the Routine maintained significantly reduced water loss at 8 h.

Apart from water content measurements, sebumetry revealed that HAS and MC individual treatments increased sebum levels over time, whereas the Routine maintained sebum levels closer to baseline at both 30 min and 8 h after application. The increase observed with MC alone is expected, given that emulsions can leave an emollient surface film and more easily interact with superficial lipids, thereby increasing the amount of sebum on the skin surface. By contrast, the HAS finding is less intuitive, as this HAS is water‐based and oil‐free. A hypothesis is that its use coincides with re‐equilibration after cleanser‐induced sebum stripping [11, 17]. Notably, the Routine remaining closer to baseline levels may suggest that combining complementary hydration mechanisms (water‐binding from HAS together with lipid replenishment from MC) reduces the need for sebum re‐equilibration after cleansing. These results are similar to observations reported in cosmetic studies evaluating formulation vehicles and skincare habits [12, 20], suggesting that a cosmetic routine sustains skin surface lipid balance rather than mitigates excessive accumulation. This lipid balance provides effective reinforcement of the skin barrier and thus skin hydration by supporting SC lipid organization without leaving the skin too oily at the end of the day, leading to better customer acceptance [10, 18].

Finally, our subjective assessments aligned with the non‐invasive instrumental outcomes: volunteers who applied the Routine reported greater immediate and long‐lasting hydration than those who received individual treatments. These results are consistent with previous clinical studies indicating that combined skincare routines improve both hydration objective measurements and subjective experience [21].

## CONCLUSION

This randomized clinical study demonstrates that while individual cosmetic products, such as HAS and MC, can provide immediate improvements in skin hydration, their combined regimen offers more sustained benefits of maintaining higher hydration levels, reducing TEWL over time, and better preserving baseline sebum levels. These results highlight the advantages of multimechanism moisturization strategies and emphasize the importance of synergy for cosmetic effectiveness.

## FUNDING INFORMATION

The work was fully sponsored by Good Molecules, LLC, a skincare brand.

## CONFLICT OF INTEREST STATEMENT

Both authors are employees of Good Molecules, LLC.

## Data Availability

The data that support the findings of this study are available from the corresponding author upon reasonable request.
